# Exploring the impact of DEP on pediatric Crohn's disease using machine learning and molecular docking methods

**DOI:** 10.3389/fped.2026.1810586

**Published:** 2026-08-18

**Authors:** Lei Zhang, Xingyu Ji, Su Ma, Huimin Chen

**Affiliations:** 1 Department of Gastroenterology, Donghai County People’s Hospital, Lianyungang, China; 2 Department of Gastroenterology, The Third Affiliated Hospital of Dali University, Dali, China

**Keywords:** Crohn's disease, diethyl phthalate, machine learning, molecular docking, pediatric

## Abstract

**Background:**

Diethyl phthalate (DEP) is a prevalent plasticizer with multiple toxic effects and high bioavailability in humans, especially children. Pediatric Crohn’s disease (PCD) is a severe chronic intestinal inflammatory disorder with a rising incidence worldwide. To date, the potential associative relationship between DEP exposure and PCD pathogenesis remains inadequately clarified, lacking systematic transcriptomic and experimental exploration.

**Methods:**

In this study, bioinformatics analysis was performed based on 682 PCD samples and 127 normal samples retrieved from multiple GEO datasets. A series of analytical strategies including differential expression analysis, weighted gene co-expression network analysis (WGCNA), compound target prediction, functional enrichment analysis, machine learning modeling, and molecular docking simulation were comprehensively utilized, followed by preliminary animal validation. All analyses in this study focused on exploring statistical correlation rather than causal relationships, given the absence of individual DEP exposure data and inherent methodological limitations.

**Results:**

Through differential analysis and WGCNA, 24 differentially expressed genes and 194 chemical targets from ChEMBL were obtained, among which 14 overlapping genes were identified. GO and KEGG analyses indicated that DEP may contribute to PCD toxicity by regulating nucleotide synthesis and amino acid metabolism. Five core genes were screened via machine learning and SHAP analysis. Animal experiments verified that PLAU protein expression in DEP, TNBS, and DEP + TNBS groups was notably higher than in the control colon tissue.

**Conclusion:**

This study systematically explores the potential associative links between DEP-related molecular dysregulation and PCD pathological alterations at the transcriptomic and tissue levels. The findings provide preliminary scientific references for understanding DEP-associated pediatric intestinal health risks and developing targeted preventive strategies for children's environmental health protection.

## 1 Introduction

Diethyl phthalate (DEP) is a common phthalate plasticizer widely applied in daily commodities and industrial supplies, covering polymer plastic products, medical appliances, personal care cosmetics and food packaging materials (1, 2). Since DEP binds physically rather than covalently with substrate materials, it is prone to leach out and diffuse into the surrounding environment. Existing monitoring studies have detected DEP residues in ambient air, indoor dust, surface water, soil and sediment, as well as multiple human biological samples such as urine and serum specimens.

Human DEP exposure mainly originates from dietary intake of contaminated food and drinking water, and hand-to-mouth contact behavior is a prominent exposure route in children. Dermal absorption from skincare products and plastic toys also contributes to total exposure burden, while inhalation exposure via volatile aerosol occupies a relatively low proportion (3). DEP ranks among the most commonly detectable phthalate compounds in human biomonitoring investigations. After entering human body, DEP is rapidly metabolized into monoethyl phthalate (MEP) in liver tissue. Both prototype DEP and its metabolite MEP can trigger oxidative stress injury, DNA damage and mitochondrial functional impairment, disturb endocrine homeostasis and steroid hormone synthesis, and interfere with nervous system development. Such adverse biological impacts pose potential health threats to children with immature immune function (1, 4).

Crohn’s disease (CD) is a chronic recurrent inflammatory bowel disorder characterized by full-thickness intestinal inflammation, which can randomly invade any segment along the digestive tract from oral cavity to anal canal. The combined action of genetic susceptibility, external environmental hazardous factors and abnormal intestinal mucosal immune response jointly participates in disease onset and progression (5). Typical clinical manifestations include intractable persistent abdominal pain, recurrent diarrhea and severe perianal lesions such as fistula and abscess, which seriously affect long-term prognosis. Pediatric and adolescent CD patients diagnosed under 18 years old usually present more aggressive clinical manifestations than adult patients. Most juvenile cases require immunosuppressant or biological agent intervention, and are accompanied by high risks of intestinal stricture, penetrating lesion, growth retardation and delayed puberty caused by persistent inflammation and nutritional malabsorption (6).

Cumulative research findings have demonstrated close correlations between environmental pollutant exposure and inflammatory bowel disease pathogenesis, whereas relevant research focusing on DEP and pediatric Crohn’s disease remains limited. Children are exposed to higher DEP level compared with adults due to frequent oral contact with plastic toys, daily use of fragranced personal care products and intake of food contaminated by migrated DEP, during the critical period of intestinal flora maturation and immune system establishment (7, 8). In the present study, multi-dimensional bioinformatics strategies including differential gene screening, weighted gene co-expression network analysis, virtual target prediction, functional enrichment annotation, machine learning biomarker identification and molecular docking simulation were adopted to analyze the potential biological association between DEP exposure and PCD pathological changes, so as to provide theoretical hints for pediatric disease prevention and environmental health management.

## 2 Methods

### 2.1 Data acquisition and processing, differential analysis

A total of three pediatric CD datasets, namely GSE57945, GSE93624, and GSE101794, were downloaded from GEO, which were all sequenced on the Illumina HiSeq 2000 (Homo sapiens). The Limma, sva, reshape2, ggplot2, and ggpubr packages were used to eliminate batch effects and merge data from GSE57945 and GSE93624, which were designated as the training set containing a total of 505 samples (428 pediatric Crohn's disease (PCD) samples and 77 normal samples). The dataset of GSE101794 was used for external validation (254 PCD, 50 normal). DEG screening was performed via the Limma package, applying cut-off criteria of |logFC| ≥ 1 and a *p*-value less than 0.05.

### 2.2 Analysis of weighted gene co-expression networks (WGCNA)

Utilizing the WGCNA package, we computed weighted correlation coefficients among genes to construct a weighted co-expression network. Prior to sample clustering, we filtered the gene expression matrix to exclude outliers. Upon determining the optimal soft threshold parameter, we generated a topological overlap matrix to aid in the development of a hierarchical clustering dendrogram. Genes with similar expression patterns were clustered within the same module, which was subsequently filtered to remove outliers, followed by sample clustering. The optimal soft threshold parameter was identified, and the topological overlap matrix was calculated to construct a hierarchical clustering dendrogram. Genes exhibiting analogous expression trends were grouped into the same module, with each module comprising at least 30 genes. Modules demonstrating a high correlation with PCD were identified. The results from the two analyses were intersected.

### 2.3 Compound structure and target prediction

The standard SMILES formula and molecular structure of DEP were retrieved via the PubChem repository (https://pubchem.ncbi.nlm.nih.gov/). DEP was searched in the chEMBL database (https://www.ebi.ac.uk/chembl/) with the filter of Homo sapiens to obtain the compound targets and related target genes. The targets of the compound were anticipated via the SwissTargetPrediction tool (http://swisstargetprediction.ch/) and SEA (https://sea.bkslab.org/) databases. The overlapping genes were identified by cross-referencing the compound-targeted genes with the differential analysis outcomes of differential analysis and WGCNA analysis.

### 2.4 Construction of toxicological regulatory network and functional pathway analysis

These overlapping genes served as the basis for building a compound-gene-disease regulatory network using Cytoscape. To understand the biological mechanism of DEP-induced pediatric Crohn's disease, the intersection genes were analyzed for Gene Ontology (GO) annotation and Kyoto Encyclopedia of Genes and Genomes (KEGG) pathway analyses were executed in R via packages like clusterProfiler, such as clusterProfiler, enrichplot, and org.Hs.eg.db, with a filter condition of *p*-value below 0.05, and the results were visualized.

### 2.5 Construction of machine learning diagnostic model and analysis

In R, 113 model combinations were written based on 12 variable selection and algorithm combinations such as Lasso, Stepglm, SVM, and glmBoost. Necessary packages such as seqinr, plyr, and randomForestSRC were installed. The training set, validation set, and intersection genes were organized to obtain the expression data of the intersection genes, and the model evaluation results were visualized through heatmaps. The model with the highest average AUC within both the training and testing datasets was designated as the optimal model. To elucidate the model's predictive mechanics, the shapviz package was utilized to conduct SHAP analysis on the specific genes of the best model, quantifying the contribution and relative importance of each gene in the model to the prediction results, and visualizing the results.

### 2.6 Analysis of immune correlations

To validate the predictive accuracy of the biomarkers for pediatric Crohn's disease, the AUC (area under the receiver operating characteristic curve) for every gene within the training set was computed, and the gene exhibiting the maximal AUC was identified as the superior predictive gene. In R, the reshape2 and ggpubr packages were used to perform correlation analysis between the best predictive gene and the corresponding differentially expressed immune cells, obtaining the correlation coefficient and *p* value, and visualizing the results. A *p* value < 0.05 indicated a significant association linking the target gene to immune cells.

### 2.7 Molecular docking studies

Structural data for the optimal predictive gene was sourced from the RCSB Protein Data Bank (https://www.rcsb.org), filtering specifically for Homo sapiens. The molecular conformation was acquired from PubChem, and the CB-Dock2 platform was employed to execute the molecular docking. A lower score indicated a more stable structure, and the result with the lowest score was selected for visualization to obtain the docking graph.

### 2.8 Animal modeling

This experiment received approval from the Ethics Committee at Donghai County People's Hospital. 6–8-week-old C57BL/6J mice, half male and half female, were divided into the control group, DEP group, TNBS group, and DEP + TNBS group. The control group was given normal saline enema. TNBS group: The dose of TNBS in the enema solution gradually increased, with 1% TNBS for sensitization on day 1, an enema of 0.75% TNBS on the 8th day, followed by 1.5% TNBS on the 15th day, and finally 2.5% TNBS enema (dissolved in 40% ethanol) on days 22, 29, and 36. The mice were sacrificed on day 43 for subsequent studies. DEP group: DEP (5 mg/kg/day) was continuously administered orally for 43 days, dissolved in corn oil. DEP + TNBS group: On the basis of the TNBS group, DEP (dissolved in corn oil) was continuously administered orally for 40 days.

### 2.9 Immunohistochemistry

Colon tissues from mice were immersed in 4% paraformaldehyde for fixation for 24 h, then dehydrated through a gradient series, and embedded in paraffin. Serial sections were prepared. The sections were dewaxed to water, stained with hematoxylin for 5 min, differentiated with alcohol hydrochloride, counterstained with eosin for 3 min, dehydrated, cleared, and mounted with neutral gum. The sections were dewaxed to water, antigen retrieval was performed with citrate buffer, endogenous peroxidase was blocked with 3% H2O2, and the primary antibody was added and incubated at 4 °C overnight. The secondary antibody was added and incubated at room temperature for 30 min. Staining was achieved using DAB, succeeded by hematoxylin counterstaining, dehydration, clearing, and mounting procedures. The tissue sections were observed under a microscope.

### 2.10 Statistical evaluation

Statistical analysis and visualization of the data were conducted using GraphPad Prism 8.02. Differences in expression were computed using the Mann–Whitney test for hub genes. A *p*-value below 0.05 was deemed statistically significant, denoted by “*” for *P* < 0.05.

## 3 Results

### 3.1 Data processing and DEGs

**Figure 1:**
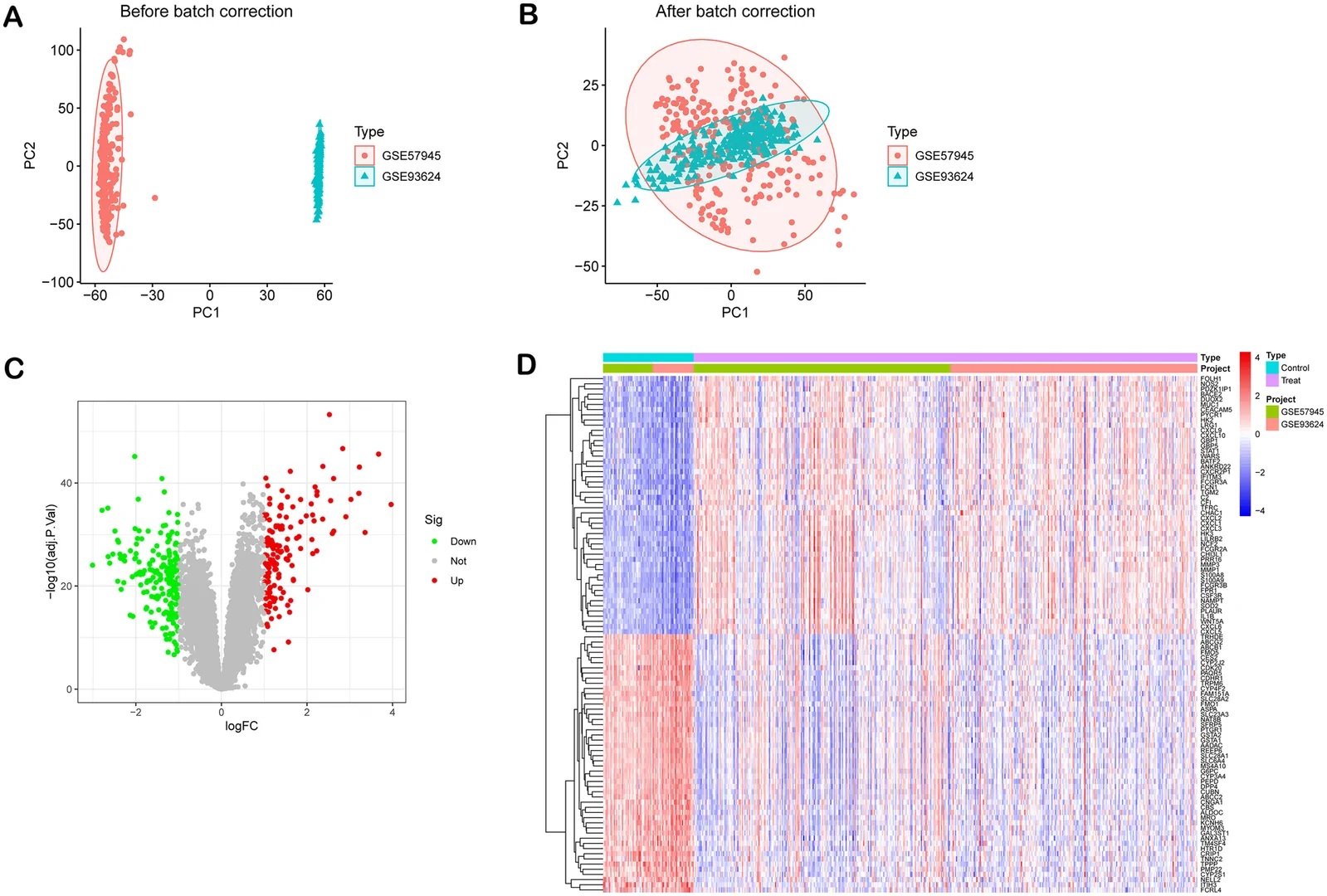
Analysis of the differences in gene expression between different datasets and the effectiveness of batch effect correction. **(A)** Distribution of samples from different experiments before batch calibration. **(B)** PCA involved randomly shuffling samples from different experiments. **(C)** Volcanic map of DEGs in the PCD dataset with |log2FC|>2. Red indicates an increase, and green indicates a decrease. **(D)** Heatmap of DEGs in the PCD dataset. Red and blue indicate upregulated and downregulated DEGs, respectively.

We combined the GSE57945 and GSE93624 datasets as the training set. It was observed that there was a batch effect between the two datasets (Figure 1A). PCA analysis was conducted to eliminate the batch effect (Figure 1B). The GSE57945 and GSE93624 datasets were merged and a differential analysis was performed to obtain significantly differentially expressed genes (DEGs), totaling 344. As shown in the volcano plot (Figure 1C), red indicates up-regulated DEGs, with a total of 149, and green indicates down-regulated DEGs, with a total of 195. The heatmap displays the top 50 genes showing the most significant up-regulation and down-regulation, with red representing up-regulated genes and blue representing down-regulated genes.

### 3.2 WGCNA analysis

**Figure 2:**
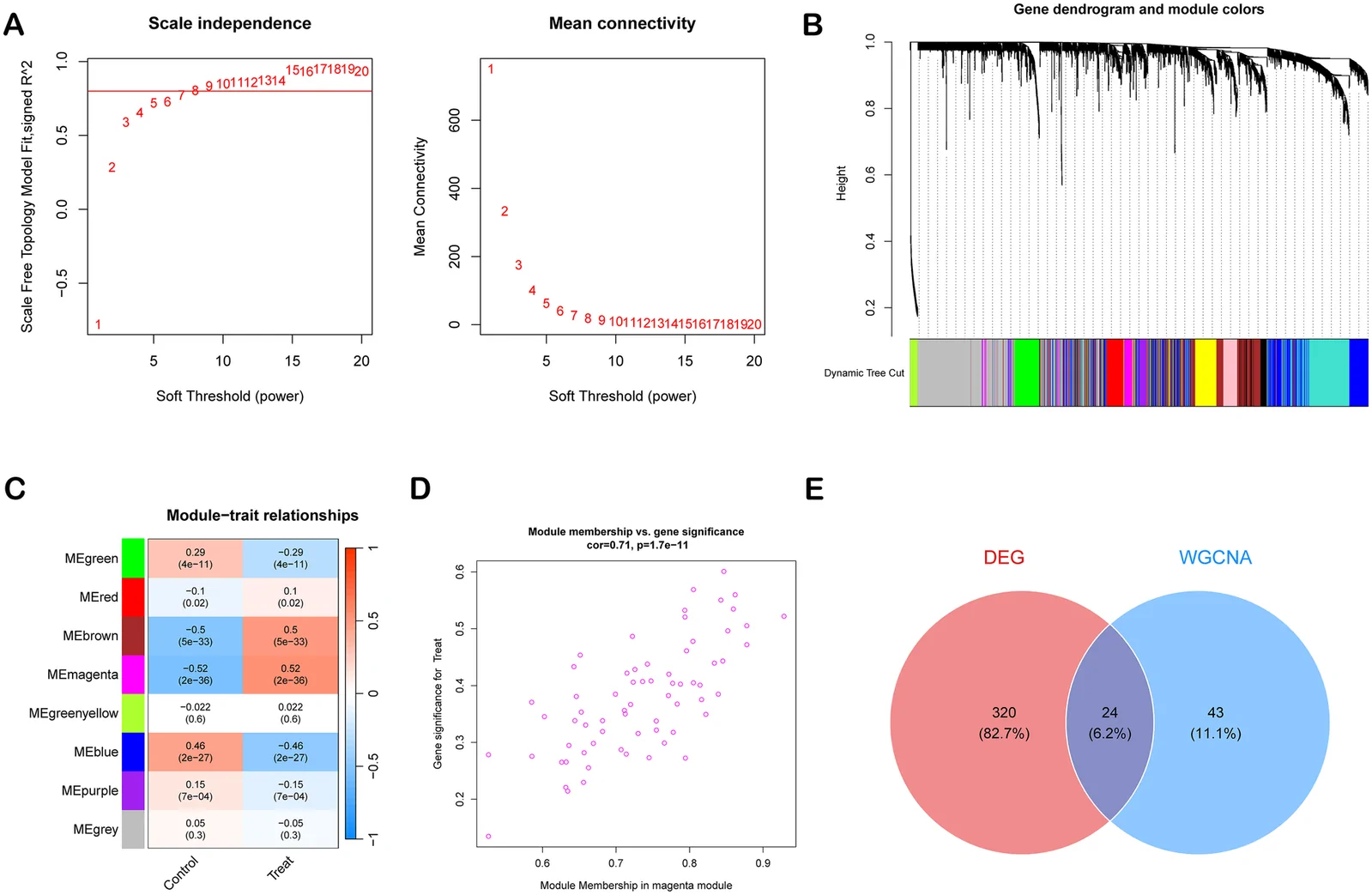
**(A)** WGCNA analysis soft threshold selection analysis. **(B)** Co-expression network clustering analysis, different colors represent different modules. **(C)** Module correlation analysis, red indicates true correlation, blue indicates negative correlation. **(D)** Scatter plot of genes in related modules. **(E)** Intersection of differential analysis and WGCNA analysis.

Through WGCNA analysis, we can obtain disease-related modules and the genes within these modules. Firstly, based on the clustering analysis of all samples, the optimal soft threshold is 11, and the optimal scale-free network is constructed (Figure 2A). Then, a co-expression network is built based on the optimal soft threshold, and the module boundaries are automatically identified using the dynamicTreeCut algorithm. Different colors represent different modules (Figure 2B). Finally, modules with similar features are merged to obtain a clinical correlation heatmap, where red indicates positive correlation, whereas blue denotes a negative one. It is evident that the magenta module is the most disease-related module (r = 0.52, *p* = 2e-36), and 67 module-related genes are obtained (Figures 2C,D). The intersection of the results from differential analysis and WGCNA analysis yields 24 genes (Figure 2E).

### 3.3 DEP target prediction

**Figure 3:**
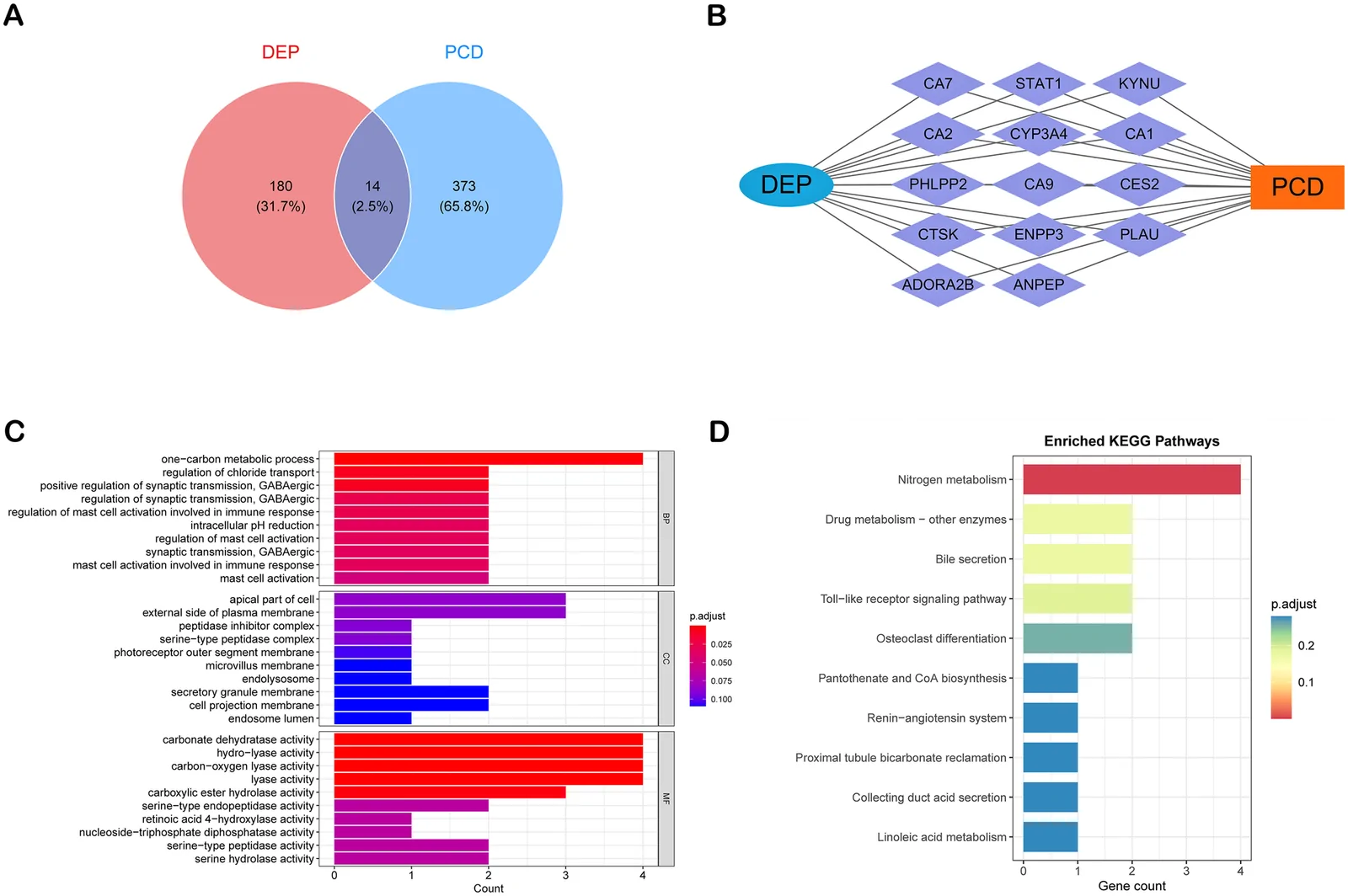
Disease-toxicant regulatory network and enrichment analysis. **(A)** DEP target genes and PCD intersection genes. **(B)** Gene disease regulatory network, blue ellipses represent compound names, purple diamonds represent gene names, and orange rectangles represent disease names. **(C, D)** Enrichment analysis of intersection genes.

The compound structure and 3D structure were acquired from the PubChem database, while both compound targets and associated predicted targets were sourced from the ChEMBL, SwissTargetPrediction and SEA repositories, yielding a total of 194 target genes. The overlap between these compound-targeted genes and those associated with the disease genes was taken to identify 14 key genes that may affect the disease (Figure 3A).

### 3.4 Regulatory network construction and enrichment analysis

Based on the above results, a regulatory network of compounds to genes to diseases was constructed in Cytoscape. Blue ellipses represent compound names, purple diamonds represent gene names, and orange rectangles represent disease names. It can be visually observed that DEP affects PCD through genes such as CA9, PHLPP2, CYP3A4, and CES2 (Figure 3B). To further investigate the potential mechanism by which DEP may affect PCD, enrichment analysis was conducted on these 14 differentially expressed genes (Figure 3C). BP enrichment analysis indicated a substantial association with one-carbon metabolic processes. One-carbon units are precursors required for purine and pyrimidine synthesis and play an important role in nucleic acid biosynthesis. The CC enrichment analysis demonstrated a strong correlation with the cell's apical region and the plasma membrane's exterior surface, indicating a close relationship with substance absorption and interaction with the external environment. The MF enrichment analysis showed a significant correlation with activity of carbonate dehydratase, hydro-lyase, carbon-oxygen lyase, and general lyase functions. These enzymes can catalyze the cleavage or formation of specific chemical bonds. KEGG enrichment analysis was performed to identify the pathways enriched by the overlapping genes (Figure 3D). Results indicated that these genes were significantly associated with nitrogen metabolism, mainly affecting the synthesis and metabolism of amino acids.

### 3.5 Machine learning diagnostic model

**Figure 4:**
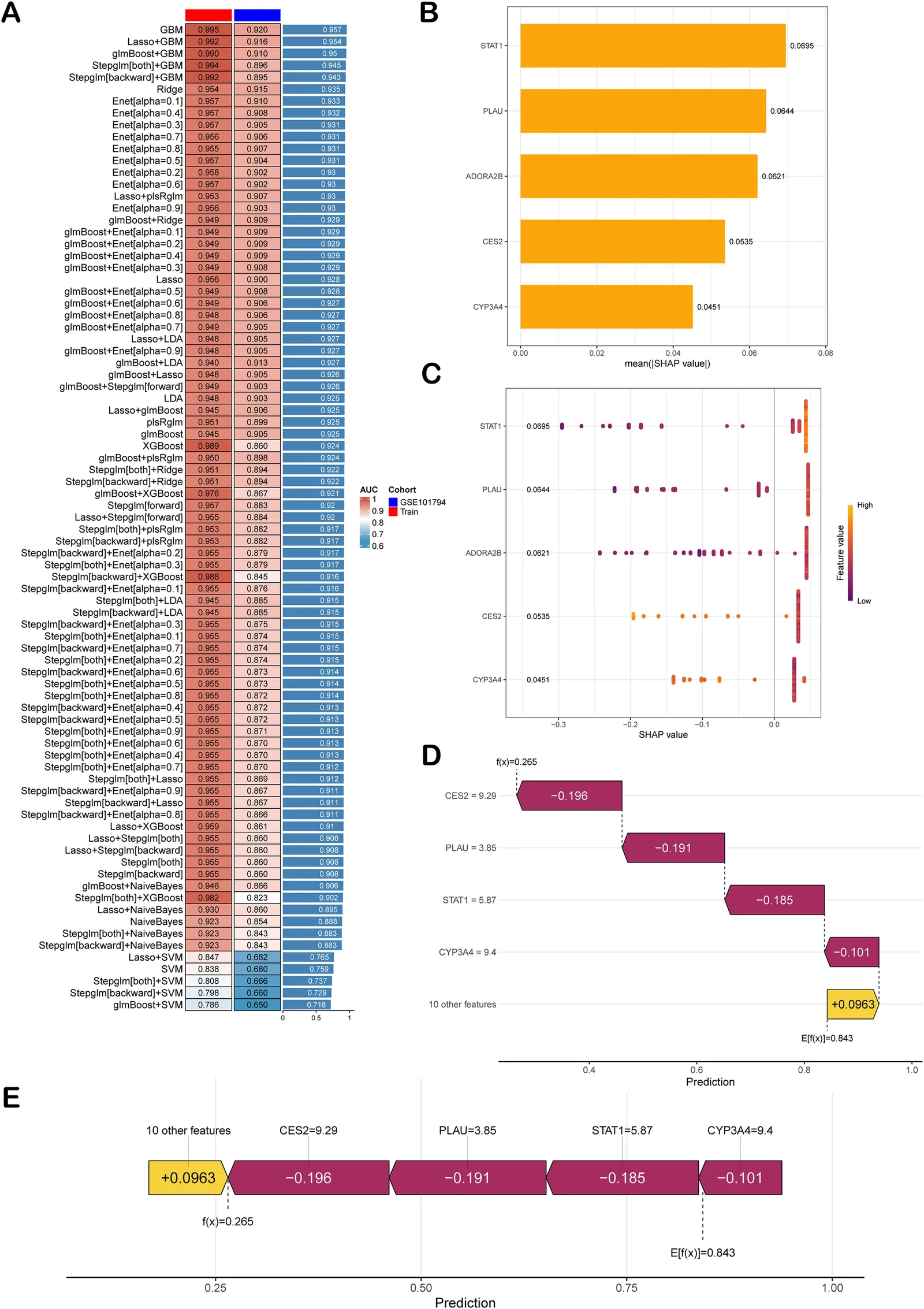
Machine learning and SHAP analysis. **(A)** 113 machine learning combinations were used to analyze the training and validation groups, and the optimal model was obtained based on the AUC value. **(B–E)** Results of SHAP analysis of the top five genes derived from the optimal model.

The combined GSE57945 and GSE93624 datasets were used as the training group, and GSE101794 as the validation group. Data analysis was conducted on the 14 intersection genes obtained above to obtain the expression levels and grouping information of the intersection genes in the training and validation groups. Through 113 machine learning combinations, the mean AUC values for every model within the validation and training datasets were computed, and the model exhibiting the maximal average value was chosen as optimal. Figure 4A displays the analysis outcomes, where the vertical axis depicts the combinations of machine learning models, the horizontal axis indicates the data sources, and the AUC values of each model in the specific data source. It can be seen that the GBM algorithm model is the best model, with an AUC score of 0.995 for the training set and 0.920 for the validation set, along with a mean value of 0.967. The GBM model identified PLAU, STAT1, ADORA2B, CES2, CYP3A4, ENPP3, KYNU, CTSK, CA9, CA7, CA1, ANPEP, CA2, and PHLPP2 as hub genes.

### 3.6 SHAP analysis

Next, we did SHAP analysis on the optimal GBM model to explain its decision-making logic and calculate feature importance based on SHAP values. SHAP values were obtained utilizing the shapviz R package. The genes were sorted by their contribution, and the top five genes were listed in descending order (Figures 4B–F). Among them, according to the bar graph, PLAU, and STAT1 contributed most significantly to the GBM performance (Figure 4B). The explanation of SHAP values follows this rule: if the SHAP value is positive, it means it has a positive contribution; otherwise, it has a negative contribution (Figure 4C). Interestingly, PLAU and STAT1 changed from purple to orange, which means that downregulation increases their SHAP values and provides protection. On the contrary, CES2 and CYP3A4 changed from orange to purple, indicating that upregulation increases their SHAP values and poses risks. The waterfall plot (Figure 4D) shows gene expression sizes, where the farther the horizontal coordinate is from zero, the greater the predictive influence of the corresponding gene. Clearly, CES2, PLAU, and STAT1 are farthest away, which means they have the largest absolute values. Finally, the force plot (Figure 4E) describes the directionality of gene effects, with longer arrows representing larger SHAP magnitudes. It is confirmed that CES2, PLAU, and STAT1 are the most predictive predictors.

### 3.7 Differential analysis of immune cells and correlation analysis

**Figure 5:**
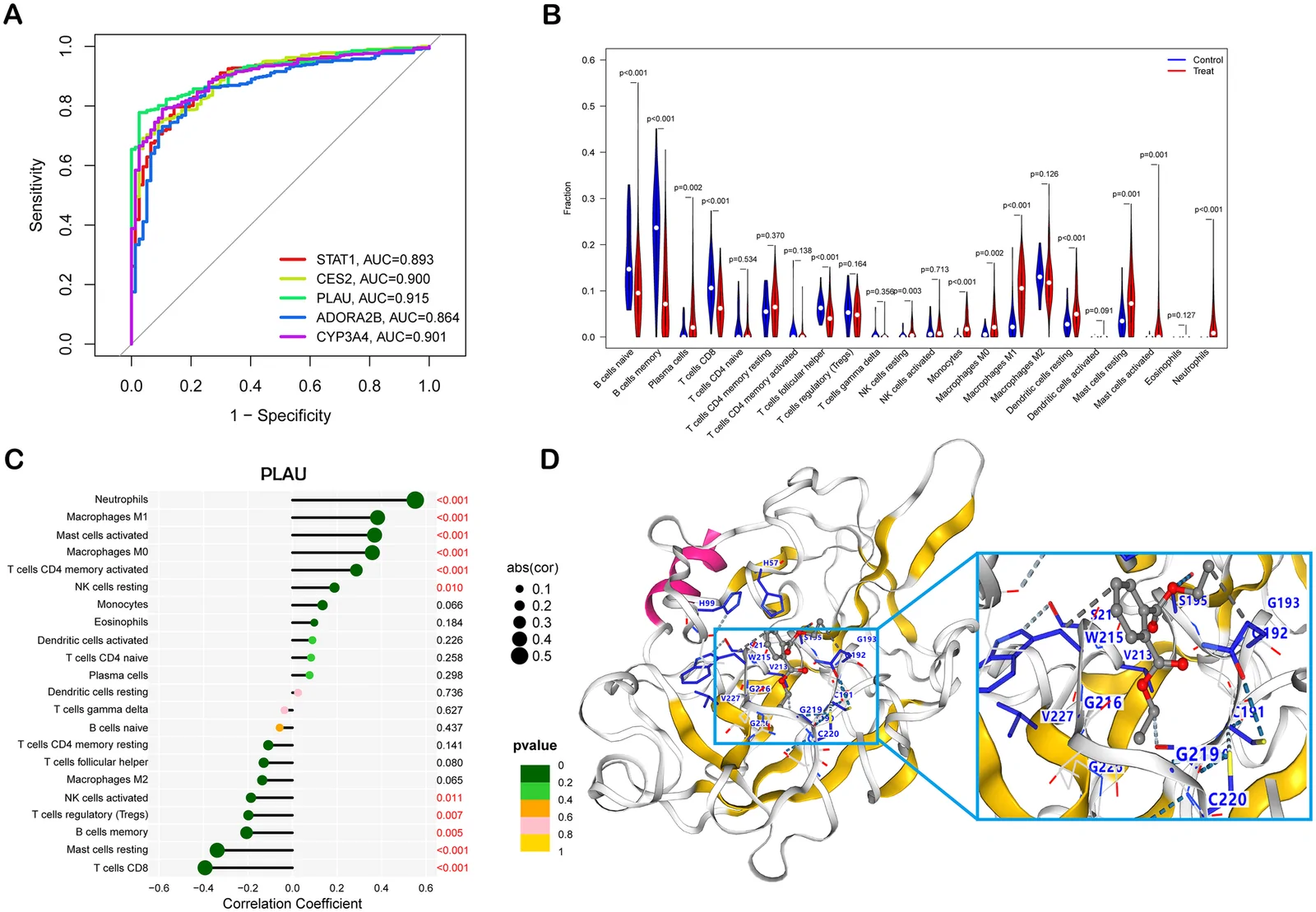
Immune-related analysis and molecular docking. **(A)** ROC curve analysis of key genes. **(B)** Immune-related analysis of the control group and disease group. **(C)** Correlation between immune cells and characteristic genes. **(D)** 3D structure molecular docking of key target genes.

PLAU, STAT1, ADORA2B, CES2, and CYP3A4 were extracted as five signature genes from SHAP-based feature selection. The diagnostic efficacy was evaluated using ROC analysis (Figure 5A). The AUC was used as an accuracy metric for control-experimental separation. All candidates exceeded the 0.85 threshold, with PLAU demonstrating the best performance (AUC 0.915). Simultaneously, parallel immune cell comparisons also revealed obvious differences between groups (Figure 5B). Among them, B cells naive, B cells memory, T cells CD8, and T cells follicular helper were notably elevated in the control cohort compared to the disease cohort. Conversely, neutrophils, mast cells activated, mast cells resting, and dendritic cells resting were significantly higher in the disease group than in the control group. Correlation analysis was conducted to examine the relationship linking immune cells with key target genes (Figure 5C). A positive correlation coefficient means a positive regulatory relationship, whereas a negative correlation coefficient signifies a negative regulatory association. PLAU showed a strong positive correlation with neutrophils, macrophages M1, mast cells activated, and macrophages M0, as well as a strong negative correlation with mast cells resting and T cells CD8, all of which were highly significant.

### 3.8 Molecular docking studies of DEP with key target genes

Protein structure data were retrieved from the RCSB Protein Data Bank to acquire the 3D structure data of PLAU. The three-dimensional structural data for key target genes and the structure data of compounds were uploaded to the CB-Dock2 database for molecular docking. A lower score indicates a more stable docking. The result with the lowest score was selected for visualization (Figure 5D).

### 3.9 Immunohistochemical verification

**Figure 6:**
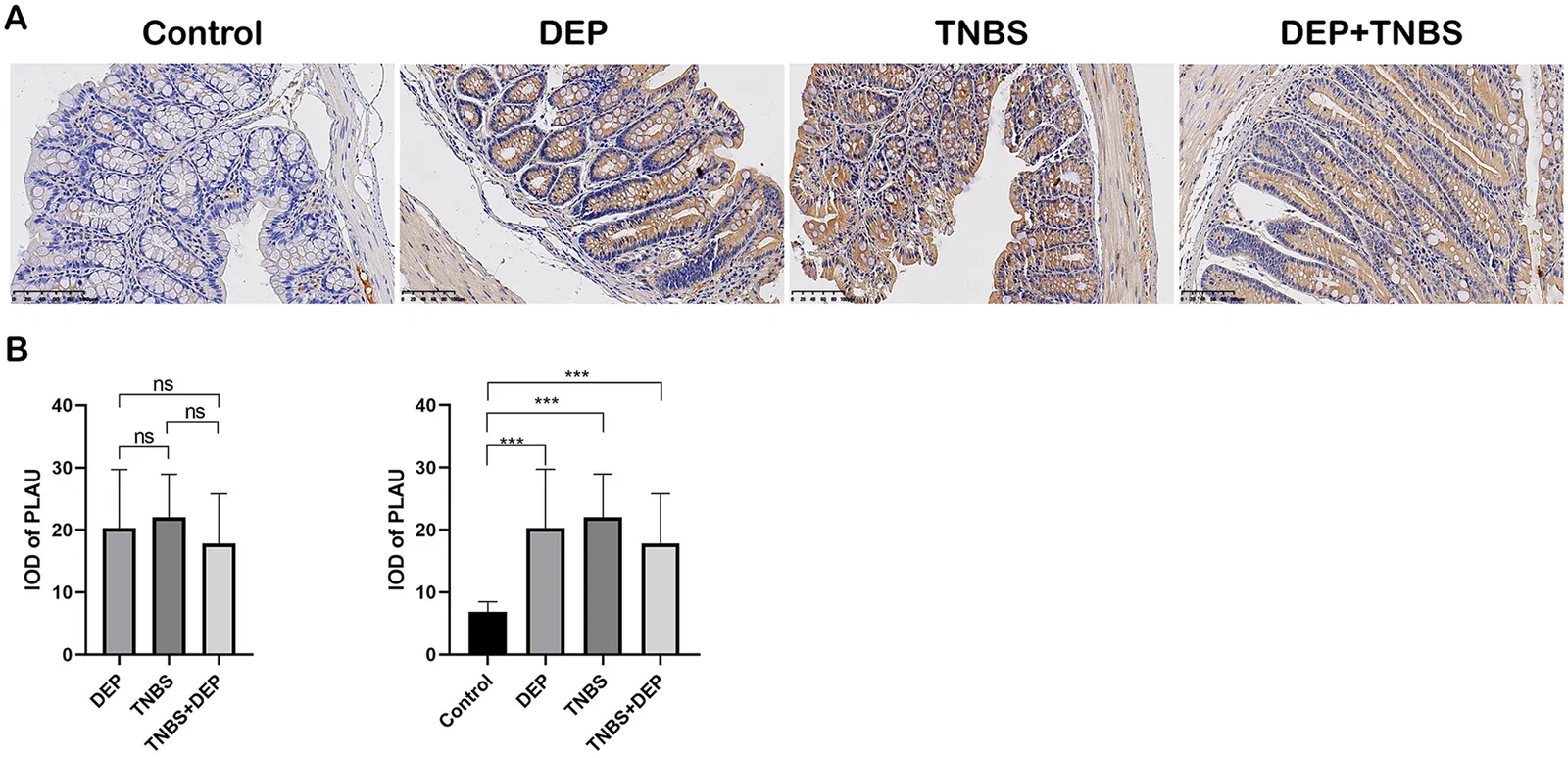
**(A)** expression of PLAU in colonic tissues of the control group, DEP group, TNBS group, and DEP + TNBS group. **(B)** Integrated optical density (IOD) values of PLAU.

PLAU was mainly expressed in the cytoplasm. The immunohistochemical results indicated that PLAU protein expression in the DEP, TNBS, and DEP + TNBS groups exceeded that found in the colon tissues of the control group. The IOD values were calculated using Image Pro Plus (IPP) software, and the analysis results were statistically significant. No statistically significant variation in expression was observed among the DEP, TNBS, and DEP + TNBS cohorts (Figure 6A,B).

## 4 Discussion

The present study integrated transcriptome data covering 682 pediatric Crohn's disease cases and 127 healthy control individuals sourced from three independent GEO datasets, adopting multi-layer bioinformatics analytical strategies combined with *in vivo* animal verification experiments, to systematically explore the potential biological association between diethyl phthalate exposure and the pathological progression of pediatric Crohn's disease. Through differential gene screening, weighted gene co-expression network analysis, compound target prediction, functional enrichment annotation, machine learning biomarker mining and molecular docking auxiliary analysis, we consistently found that the alteration of one-carbon metabolism and nitrogen metabolism pathways presented prominent correlation with the occurrence and development of PCD. A panel of hub genes including PLAU, STAT1, ADORA2B, CES2 and CYP3A4 were identified as key molecular markers closely linked to DEP exposure and disease phenotype changes. Subsequent murine colitis model experiments further provided phenotypic evidence that DEP intervention was accompanied with elevated PLAU protein expression in intestinal tissues, which corroborated the analytical outcomes acquired from transcriptome data.

From the perspective of immunometabolism, the metabolic pathway variations screened in this research are highly consistent with existing academic understandings of inflammatory bowel disease pathogenesis. As reported in relevant studies, one-carbon metabolism acts as an indispensable metabolic hub inside cells, whose metabolic flux directly governs the *de novo* synthesis process of purine and pyrimidine nucleotides (9, 10). The disturbed homeostasis of one-carbon metabolism will break the balance of T lymphocyte subset differentiation, drive CD4+ T cells to differentiate toward pro-inflammatory Th1 phenotype, and ultimately trigger persistent amplified inflammatory response in intestinal mucosa. Meanwhile, the blocked salvage metabolic pathway will weaken intracellular antioxidant defense capacity, hinder the clearance of reactive oxygen species, continuously activate NF-*κ*B mediated inflammatory signaling cascade, remodel local immune microenvironment and progressively aggravate intestinal tissue inflammatory damage.

Apart from carbon-related metabolic disorder, abnormal nitrogen metabolism also occupies an important position in intestinal inflammatory lesions. In inflammatory infiltrated lamina propria, nitrogen oxide generated by activated macrophages and neutrophils can react with superoxide anion to form peroxynitrite, a highly oxidative toxic substance (11, 12). Such substance will trigger lipid peroxidation damage, irreversible protein nitration modification and mitochondrial structural dysfunction of intestinal epithelial cells, collectively impairing intestinal barrier integrity. Additionally, nitrogen metabolic imbalance is tightly bound with intestinal flora composition disorder. Proliferation of urease-rich bacteria will elevate intestinal nitrogen absorption level, reduce the proportion of beneficial Firmicutes, and form a vicious cycle that sustains chronic inflammatory state in intestinal tract (13). This series of changes further indicates metabolic disturbance can serve as important associated factors of PCD deterioration.

Among all screened pivotal genes, PLAU exhibits complex and dual biological functions worthy of in-depth discussion. Under physiological steady state, PLAU participates in extracellular matrix remodeling, tissue damage repair and orderly proteolysis process, maintaining normal intestinal tissue structure and physiological function (14, 15). Whereas in pathological inflammatory environment, evident upregulation of PLAU expression was observed both in bioinformatics analysis and immunohistochemical detection results. Existing research has confirmed that abnormally increased PLAU can regulate GPX4 expression level through transcriptional and post-transcriptional pathways, thereby modulating cell ferroptosis sensitivity (16, 17). Combined with clinical research evidence that active Crohn's disease patients possess lower macrophage GPx1 activity and higher ferroptosis susceptibility, our findings further extend this pathological rule to pediatric population, inferring PLAU may serve as an upstream associated factor mediating intestinal cell ferroptosis and tissue fibrosis lesions (18–20).

Immune correlation analysis also revealed intimate links between PLAU expression and diverse immune cell infiltration characteristics. PLAU level showed strong positive correlation with neutrophils, M1-type macrophages and activated mast cells, these cell subsets are widely recognized as core inflammatory participants that destroy mucosal barrier, induce granuloma formation and promote tissue fibrosis (21–25). Activated mast cell tryptase can further activate plasmin via PLAU-dependent pathway, and activated plasmin in turn accelerates extracellular matrix degradation, stimulates pro-inflammatory cytokines TGF-*β* and IL-1β release, and recruits more inflammatory leukocytes to lesion sites, eventually forming self-amplifying inflammatory feedback loop (23, 24). Comprehensively, PLAU is not merely a simple disease diagnostic biomarker, but a critical intermediate molecule that correlates DEP exposure stimulation, intracellular metabolic disorder, innate immune activation and intestinal tissue remodeling process.

Machine learning modeling constructed in this work effectively mined characteristic gene signatures distinguishing PCD and normal samples, though relatively high AUC value of training set reflected inherent model fitting tendency and potential overfitting risk, external validation results still verified the basic predictive potential of core genes. Molecular docking simulation reflected favorable binding affinity between DEP and PLAU protein, which can be taken as auxiliary suggestive evidence of molecular interaction possibility, rather than definitive mechanistic proof of action mode.

## 5 Conclusion

In conclusion, this study systematically analyzed the transcriptomic characteristics of pediatric Crohn's disease based on multi-center GEO datasets and preliminarily explored the potential associative relationship between DEP-related molecular disturbance and PCD metabolic disorder and intestinal inflammatory response. The results indicated that DEP-associated gene dysregulation was statistically correlated with abnormal one-carbon and nitrogen metabolism in intestinal tissues, and PLAU was identified as a core biomarker with high correlation with intestinal immune microenvironment disorder after DEP intervention. Animal experiments preliminarily verified the up-regulated expression trend of PLAU protein in DEP-intervened inflammatory intestinal tissues. This study provides preliminary exploratory evidence and reference value for understanding the potential correlation between environmental DEP pollution and pediatric intestinal inflammatory injury.

However, this study still exists several inherent limitations. Firstly, fixed single DEP dosage was adopted in animal experiment, which cannot fully simulate low-dose long-term cumulative exposure characteristics of real human population. Secondly, all bioinformatics analytical results only reflect statistical association characteristics, and sufficient evidence supporting definite causal regulatory relationship has not been obtained. Thirdly, population epidemiological exposure data and clinical longitudinal observation samples are still lacking, further external verification is required to confirm research conclusion applicability.

## Data Availability

The original contributions presented in the study are included in the article/Supplementary Material, further inquiries can be directed to the corresponding author.
